# Lower bounds on trees and unicyclic graphs with respect to the misbalance rodeg index

**DOI:** 10.1016/j.heliyon.2024.e41235

**Published:** 2024-12-17

**Authors:** Nasrin Dehgardi, Mahdieh Azari, Yilun Shang

**Affiliations:** a Department of Mathematics and Computer Science, Sirjan University of Technology, Sirjan, Iran; b Department of Mathematics, Kazerun Branch, Islamic Azad University, P. O. Box: 73135-168, Kazerun, Iran; c Department of Computer and Information Sciences, Northumbria University, Newcastle, NE1 8ST, UK

**Keywords:** 05C05, 05C07, 05C09, 05C35, Graph irregularity, Misbalance rodeg index, Trees, Unicyclic graphs, Sharp bounds

## Abstract

The Misbalance Rodeg (*MR*) index stands out among the 148 discrete Adriatic indices demonstrating considerable predictive capabilities in evaluations carried out by the International Academy of Mathematical Chemistry. This index excels particularly in forecasting both the enthalpy and the standard enthalpy of vaporization for octane isomers. Despite its significant chemical applicability, the *MR* index has not been extensively explored in the literature. One objective of this study is to highlight the importance of this graph invariant to the mathematical chemistry community by examining various mathematical properties associated with it. Our investigation specifically aims to ascertain the minimal values of the *MR* index for all trees and unicyclic graphs with a given order and maximum vertex degree. Additionally, we extend our analysis to molecular trees and provide a characterization of the respective minimal trees and unicyclic graphs.

## 1 Introduction

Consider a simple graph $G=(V^{G},E^{G})$, where $V^{G}$ represents the set of vertices and $E^{G}$ represents the set of edges of *G*. For a vertex $\omega \in V^{G}$, the open neighborhood $N_{\omega }^{G}$ is defined as the collection of vertices that are directly connected to *ω* by an edge. Mathematically, this can be expressed as $N_{\omega }^{G}=\{v\in V^{G}:\omega v\in E^{G}\}$. The degree $d_{\omega }^{G}$ of vertex *ω* is the number of vertices in its open neighborhood. The distance between two vertices *ω* and *v*, represented as $d_{\left(\omega ,v\right)}^{G}$, is considered as the length of a shortest path connecting them within the graph.

In a tree (also referred to as an acyclic graph), a branching vertex is considered a vertex with a degree greater than 2, while a leaf is a vertex with a degree of 1. A rooted tree is a specific type of tree in which one vertex is designated as the root, establishing a hierarchy among the vertices. A chemical tree (also refereed to as a molecular tree) is a type of tree in which the maximum degree is four or less.

A graph invariant is a property inherent to a graph's structure that does not depend on how the graph is represented or how its vertices and edges are labeled. In the field of chemistry, these invariants are referred to as topological indices. Their significance has notably increased due to their application in Quantitative Structure-Activity/Property Relationships (QSAR/QSPR) [26]. Among these topological indices, some are classified as bond additive, meaning they can be calculated by summing the contributions from individual edges.

In 2010, Vukićević and Gašperov [28] introduced a collection known as the Discrete Adriatic indices, which consists of 148 bond additive invariants. These invariants have demonstrated strong predictive capabilities in evaluations conducted by the International Academy of Mathematical Chemistry (IAMC). They are designed for easy implementation and can be incorporated into existing molecular modeling software. Among the 148 Adriatic indices, certain indices have shown exceptional efficiency. One notable example is the Misbalance Rodeg index (shortened to *MR* index), which is defined for a graph *G* as follows:

$$MR(G)=\sum_{\omega v\in E^{G}}\left|\sqrt{d_{\omega }^{G}}-\sqrt{d_{v}^{G}}\right|.$$

The Misbalance Rodeg index has demonstrated robust predictive capabilities, particularly concerning the standard enthalpy of vaporization and the enthalpy of vaporization for isomers of octane, as established in previous studies [28].

A graph *G* is considered regular if each vertex has the same degree. If the degrees of the vertices differ, then *G* is classified as irregular. The necessary and sufficient condition for a non-negative topological index *TI* to serve as a measure of irregularity in the graph *G* is that TI(G)=0 if and only if *G* is regular. Measures of graph irregularity play a prominent role in chemistry and network theory [5]. In the last decade, several measures of graph irregularity have been introduced, including the Albertson irregularity and total irregularity indices [1], [4], [8], [29], the first irregularity Sombor index [9], [15], the sigma index [2], [13], the Misbalance Prodeg index [17], and degree deviation [6], [20]. Irregularity measures have been well studied in the literature [3], [11], [23], [24]. Given that the conditions for measuring irregularity apply to the *MR* index, it is justifiable to consider this index a valid measure of graph irregularity.

Although more than a decade has passed since the introduction of the *MR* index, relatively little research has been conducted on this index thus far. Anuradha et al. [7] assessed the *MR* index alongside various discrete Adriatic indices and their corresponding entropy invariants to forecast specific physicochemical features of curcumin-conjugated PAMAM dendrimers resulting from two growth steps. Redzepović [22] compared the predictive potential of several vertex-degree-based indices, including the *MR* index. Tharmalingam et al. [25] computed the *MR* index of hexabenzocoronene, while Kulli et al. [16] studied the *MR* index across various graph classes such as hyper-cubes, generalized Petersen graphs, Cartesian products, grids, tori, cylinders, lollipops, and Harary graphs. Lokesha et al. [18] computed several discrete Adriatic indices for certain classes of derived graphs using some graph operators namely line, subdivision, semi-total, total, jump, and para-line graphs. Vasilyev and Stevanović[27] applied MathChem software to calculate the *MR* index along with several other discrete Adriatic indices. Milovanović et al. [19] proposed a modified version of the *MR* index and investigated some extremal problems related to this invariant. Kulli [14] introduced a leap version of the discrete Adriatic indices that includes the *MR* index and computed these invariants for polycyclic aromatic hydrocarbons. Recent studies on other misbalance indices can be found in [10], [12], [17], [21].

In this paper, we intend to examine some mathematical properties of the *MR* index. We focus on two specific families of graphs: trees and unicyclic graphs, where both the order and maximum degree are fixed. Our goal is to provide sharp lower bounds on the *MR* index within these two families and to characterize the graphs that achieve these lower bounds.

## 2 Lower bounds on trees with fixed order and maximum degree

**Figure 1:**
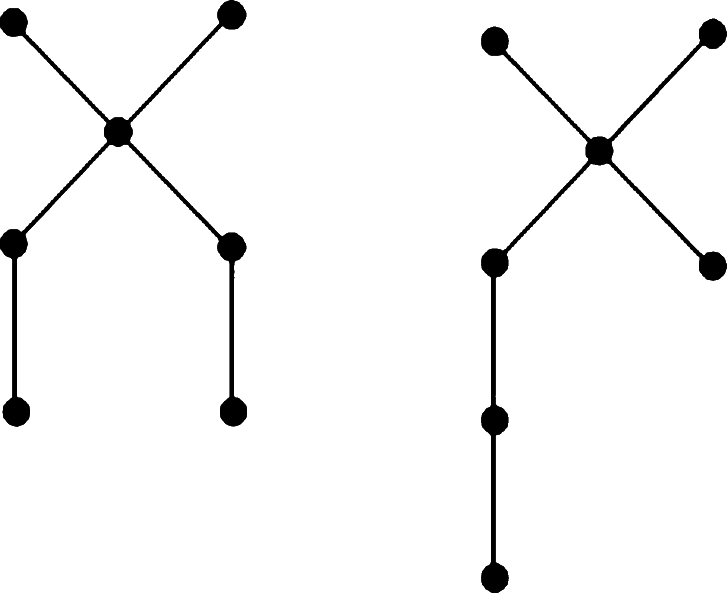
Spider graphs with *υ* = 7 vertices and Δ = 4.

In this section, we derive lower bounds for trees concerning the *MR* index. The notation $T_{\upsilon }^{\Delta }$ represents the set of all trees with *υ* vertices and a maximum degree Δ. A spider is defined as a tree that has at most one branching vertex, referred to as its center (see Fig. 1). In each spider, the paths that connect the center to the leaves are termed the legs of the spider. A path is categorized as a spider having one leg or two legs.

Lemma 1 *Assume that T represents a rooted tree in*$T_{\upsilon }^{\Delta }$*, with its root located at vertex ω, which has a degree of* Δ*. If T contains a branching vertex other than ω, then there is another tree* $T^{\prime }$ *within* $T_{\upsilon }^{\Delta }$ *in such a way that* $MR(T^{\prime })<MR(T)$*.*

Proof Let *ϑ* be a branching vertex in the tree *T*, distinct from the root vertex *ω*, that is farthest from *ω* among all other branching vertices of *T* and let $d_{\vartheta }^{T}=\lambda \geq 3$. Assume that the neighborhood of *ϑ* is defined as $N_{\vartheta }^{T}=\{\vartheta_{1},\vartheta_{2},\ldots ,\vartheta_{\lambda }\}$, where $\vartheta_{\lambda }$ lies on the path from *ω* to *ϑ*. Thus, for each vertex $\vartheta_{i}$, (where 1≤i≤λ−1), it holds that $d_{\vartheta_{i}}^{T}\in \{1,2\}$. Consequently, there are three cases to consider.

**Case 1.**$N_{\vartheta }^{T}$ has at least two leaves.

**Figure 2:**
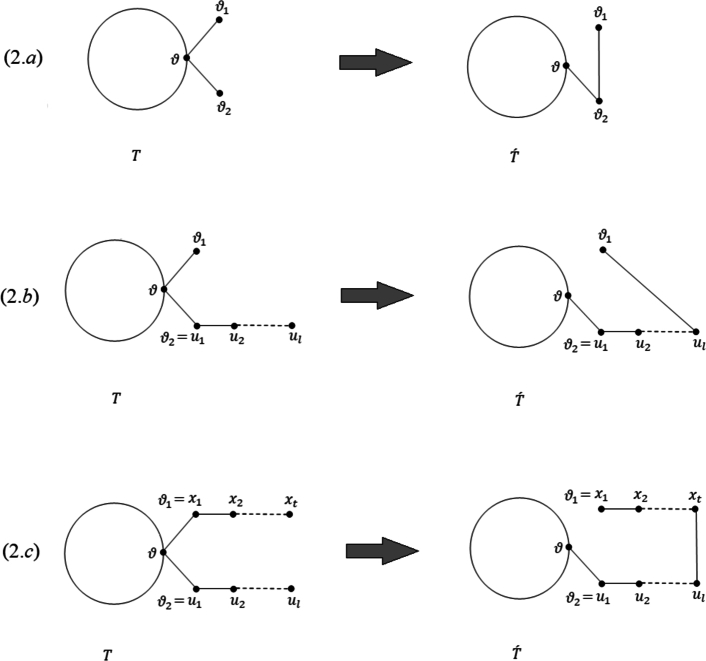
(2.*a*) The transformation applied in Case 1 of Lemma 1, (2.*b*) the transformation applied in Case 2 of Lemma 1, (2.*c*) the transformation applied in Case 3 of Lemma 1.

Suppose that $\vartheta_{1}$ and $\vartheta_{2}$ are two leaves. Set $T^{\prime }=(T-\{\vartheta \vartheta_{1}\})+\{\vartheta_{1}\vartheta_{2}\}$ (see Fig. (2.*a*)). Then,

$$MR(T)-MR(T^{\prime })=\sum_{uv\in E^{T}}|\sqrt{d_{u}^{T}}-\sqrt{d_{v}^{T}}|-\sum_{uv\in E^{T^{\prime }}}|\sqrt{d_{u}^{T^{\prime }}}-\sqrt{d_{v}^{T^{\prime }}}|=|\sqrt{d_{\vartheta }^{T}}-\sqrt{d_{\vartheta_{1}}^{T}}|+|\sqrt{d_{\vartheta }^{T}}-\sqrt{d_{\vartheta_{2}}^{T}}|+|\sqrt{d_{\vartheta }^{T}}-\sqrt{d_{\vartheta_{\lambda }}^{T}}|+\sum_{i=3}^{\lambda -1}|\sqrt{d_{\vartheta }^{T}}-\sqrt{d_{\vartheta_{i}}^{T}}|-|\sqrt{d_{\vartheta_{2}}^{T^{\prime }}}-\sqrt{d_{\vartheta_{1}}^{T^{\prime }}}|-|\sqrt{d_{\vartheta }^{T^{\prime }}}-\sqrt{d_{\vartheta_{2}}^{T^{\prime }}}|-|\sqrt{d_{\vartheta }^{T^{\prime }}}-\sqrt{d_{\vartheta_{\lambda }}^{T^{\prime }}}|-\sum_{i=3}^{\lambda -1}|\sqrt{d_{\vartheta }^{T^{\prime }}}-\sqrt{d_{\vartheta_{i}}^{T^{\prime }}}|=2(\sqrt{\lambda }-1)+|\sqrt{\lambda }-\sqrt{d_{\vartheta_{\lambda }}^{T}}|+\sum_{i=3}^{\lambda -1}(\sqrt{\lambda }-\sqrt{d_{\vartheta_{i}}^{T}})-(\sqrt{2}-1)-(\sqrt{\lambda -1}-\sqrt{2})-|\sqrt{\lambda -1}-\sqrt{d_{\vartheta_{\lambda }}^{T}}|-\sum_{i=3}^{\lambda -1}(\sqrt{\lambda -1}-\sqrt{d_{\vartheta_{i}}^{T}})=2\sqrt{\lambda }-\sqrt{\lambda -1}-1+(\lambda -3)(\sqrt{\lambda }-\sqrt{\lambda -1})+|\sqrt{\lambda }-\sqrt{d_{\vartheta_{\lambda }}^{T}}|-|\sqrt{\lambda -1}-\sqrt{d_{\vartheta_{\lambda }}^{T}}|.$$

If $\lambda >d_{\vartheta_{\lambda }}^{T}$, then

$$|\sqrt{\lambda }-\sqrt{d_{\vartheta_{\lambda }}^{T}}|-|\sqrt{\lambda -1}-\sqrt{d_{\vartheta_{\lambda }}^{T}}|=(\sqrt{\lambda }-\sqrt{d_{\vartheta_{\lambda }}^{T}})-(\sqrt{\lambda -1}-\sqrt{d_{\vartheta_{\lambda }}^{T}})=\sqrt{\lambda }-\sqrt{\lambda -1},$$

and

$$MR(T)-MR(T^{\prime })=2\sqrt{\lambda }-\sqrt{\lambda -1}-1+(\lambda -2)(\sqrt{\lambda }-\sqrt{\lambda -1})>0.$$

Now if $\lambda \leq d_{\vartheta_{\lambda }}^{T}$, then

$$|\sqrt{\lambda }-\sqrt{d_{\vartheta_{\lambda }}^{T}}|-|\sqrt{\lambda -1}-\sqrt{d_{\vartheta_{\lambda }}^{T}}|=(\sqrt{d_{\vartheta_{\lambda }}^{T}}-\sqrt{\lambda })-(\sqrt{d_{\vartheta_{\lambda }}^{T}}-\sqrt{\lambda -1})=\sqrt{\lambda -1}-\sqrt{\lambda },$$

and

$$MR(T)-MR(T^{\prime })=\sqrt{\lambda }-1+(\lambda -3)(\sqrt{\lambda }-\sqrt{\lambda -1})>0.$$

**Case 2.**$N_{\vartheta }^{T}$ includes just one leaf.

Denote the leaf in $N_{\vartheta }^{T}$ by $\vartheta_{1}$ and assume that the path $\vartheta u_{1}u_{2}\ldots u_{l}$ (where l≥2) exists in *T* with $u_{1}=\vartheta_{2}$. Define the tree $T^{\prime }$ as $T^{\prime }=(T-\{\vartheta \vartheta_{1}\})+\{\vartheta_{1}u_{l}\}$ (see Fig. (2.*b*)). Then,

$$MR(T)-MR(T^{\prime })=\sum_{uv\in E^{T}}|\sqrt{d_{u}^{T}}-\sqrt{d_{v}^{T}}|-\sum_{uv\in E^{T^{\prime }}}|\sqrt{d_{u}^{T^{\prime }}}-\sqrt{d_{v}^{T^{\prime }}}|=|\sqrt{d_{\vartheta }^{T}}-\sqrt{d_{\vartheta_{1}}^{T}}|+|\sqrt{d_{u_{l}}^{T}}-\sqrt{d_{u_{l-1}}^{T}}|+|\sqrt{d_{\vartheta }^{T}}-\sqrt{d_{\vartheta_{\lambda }}^{T}}|+\sum_{i=3}^{\lambda -1}|\sqrt{d_{\vartheta }^{T}}-\sqrt{d_{\vartheta_{i}}^{T}}|-|\sqrt{d_{\vartheta_{1}}^{T^{\prime }}}-\sqrt{d_{u_{l}}^{T^{\prime }}}|-|\sqrt{d_{u_{l}}^{T^{\prime }}}-\sqrt{d_{u_{l-1}}^{T^{\prime }}}|-|\sqrt{d_{\vartheta }^{T^{\prime }}}-\sqrt{d_{\vartheta_{\lambda }}^{T^{\prime }}}|-\sum_{i=3}^{\lambda -1}|\sqrt{d_{\vartheta }^{T^{\prime }}}-\sqrt{d_{\vartheta_{i}}^{T^{\prime }}}|=(\sqrt{\lambda }-1)+(\sqrt{2}-1)+|\sqrt{\lambda }-\sqrt{d_{\vartheta_{\lambda }}^{T}}|+\sum_{i=3}^{\lambda -1}(\sqrt{\lambda }-\sqrt{d_{\vartheta_{i}}^{T}})-(\sqrt{2}-1)-(\sqrt{2}-\sqrt{2})-|\sqrt{\lambda -1}-\sqrt{d_{\vartheta_{\lambda }}^{T}}|-\sum_{i=3}^{\lambda -1}(\sqrt{\lambda -1}-\sqrt{d_{\vartheta_{i}}^{T}})=\sqrt{\lambda }-1+(\lambda -3)(\sqrt{\lambda }-\sqrt{\lambda -1})+|\sqrt{\lambda }-\sqrt{d_{\vartheta_{\lambda }}^{T}}|-|\sqrt{\lambda -1}-\sqrt{d_{\vartheta_{\lambda }}^{T}}|.$$

If $\lambda >d_{\vartheta_{\lambda }}^{T}$, then

$$|\sqrt{\lambda }-\sqrt{d_{\vartheta_{\lambda }}^{T}}|-|\sqrt{\lambda -1}-\sqrt{d_{\vartheta_{\lambda }}^{T}}|=(\sqrt{\lambda }-\sqrt{d_{\vartheta_{\lambda }}^{T}})-(\sqrt{\lambda -1}-\sqrt{d_{\vartheta_{\lambda }}^{T}})=\sqrt{\lambda }-\sqrt{\lambda -1},$$

and

$$MR(T)-MR(T^{\prime })=\sqrt{\lambda }-1+(\lambda -2)(\sqrt{\lambda }-\sqrt{\lambda -1})>0.$$

Now if $\lambda \leq d_{\vartheta_{\lambda }}^{T}$, then

$$|\sqrt{\lambda }-\sqrt{d_{\vartheta_{\lambda }}^{T}}|-|\sqrt{\lambda -1}-\sqrt{d_{\vartheta_{\lambda }}^{T}}|=(\sqrt{d_{\vartheta_{\lambda }}^{T}}-\sqrt{\lambda })-(\sqrt{d_{\vartheta_{\lambda }}^{T}}-\sqrt{\lambda -1})=\sqrt{\lambda -1}-\sqrt{\lambda },$$

and

$$MR(T)-MR(T^{\prime })=\sqrt{\lambda -1}-1+(\lambda -3)(\sqrt{\lambda }-\sqrt{\lambda -1})>0.$$

**Case 3.** Let $d_{\vartheta_{i}}^{T}=2$, for 1≤i≤λ.

Let $\vartheta x_{1}x_{2}\ldots x_{t}$ and $\vartheta u_{1}u_{2}\ldots u_{l}$ represent two paths in *T*, with $x_{1}=\vartheta_{1}$, $u_{1}=\vartheta_{2}$, and t,l≥2. Assume that $T^{\prime }=(T-\{\vartheta \vartheta_{1}\})+\{\vartheta_{1}u_{l}\}$ (see Fig. (2.*c*)). Then,

$$MR(T)-MR(T^{\prime })=\sum_{uv\in E^{T}}|\sqrt{d_{u}^{T}}-\sqrt{d_{v}^{T}}|-\sum_{uv\in E^{T^{\prime }}}|\sqrt{d_{u}^{T^{\prime }}}-\sqrt{d_{v}^{T^{\prime }}}|=|\sqrt{d_{\vartheta }^{T}}-\sqrt{d_{\vartheta_{1}}^{T}}|+|\sqrt{d_{u_{l}}^{T}}-\sqrt{d_{u_{l-1}}^{T}}|+|\sqrt{d_{\vartheta }^{T}}-\sqrt{d_{\vartheta_{\lambda }}^{T}}|+\sum_{i=3}^{\lambda -1}|\sqrt{d_{\vartheta }^{T}}-\sqrt{d_{\vartheta_{i}}^{T}}|-|\sqrt{d_{\vartheta_{1}}^{T^{\prime }}}-\sqrt{d_{u_{l}}^{T^{\prime }}}|-|\sqrt{d_{u_{l}}^{T^{\prime }}}-\sqrt{d_{u_{l-1}}^{T^{\prime }}}|-|\sqrt{d_{\vartheta }^{T^{\prime }}}-\sqrt{d_{\vartheta_{\lambda }}^{T^{\prime }}}|-\sum_{i=3}^{\lambda -1}|\sqrt{d_{\vartheta }^{T^{\prime }}}-\sqrt{d_{\vartheta_{i}}^{T^{\prime }}}|=(\sqrt{\lambda }-\sqrt{2})+(\sqrt{2}-1)+|\sqrt{\lambda }-\sqrt{d_{\vartheta_{\lambda }}^{T}}|+\sum_{i=3}^{\lambda -1}(\sqrt{\lambda }-\sqrt{d_{\vartheta_{i}}^{T}})-2(\sqrt{2}-\sqrt{2})-|\sqrt{\lambda -1}-\sqrt{d_{\vartheta_{\lambda }}^{T}}|-\sum_{i=3}^{\lambda -1}(\sqrt{\lambda -1}-\sqrt{d_{\vartheta_{i}}^{T}})=\sqrt{\lambda }-1+(\lambda -3)(\sqrt{\lambda }-\sqrt{\lambda -1})+|\sqrt{\lambda }-\sqrt{d_{\vartheta_{\lambda }}^{T}}|-|\sqrt{\lambda -1}-\sqrt{d_{\vartheta_{\lambda }}^{T}}|.$$

In a manner similar to Case 2, we have that $MR(T)-MR(T^{\prime })>0$. □

The following lemma is derived from the transformations outlined in the proof of the previous lemma.

Lemma 2 *Assume that T represents a tree in* $T_{\upsilon }^{\Delta }$ *, with its root located at vertex ω, which has a degree of λ, where* 1≤λ≤Δ *. If T contains a branching vertex other than ω, then there is another tree* $T^{\prime }$ *within* $T_{\upsilon }^{\Delta }$ *in such a way that* $MR(T^{\prime })<MR(T)$ *.*

Below is one of our main theorems:

Theorem 3 *For each tree T within* $T_{\upsilon }^{\Delta }$ *, we have*

$$MR(T)\geq \Delta (\sqrt{\Delta }-1),$$

*with equality iff T is a spider graph.*

Proof Let $T^{\prime }$ be a tree in $T_{\upsilon }^{\Delta }$ that satisfies the inequality $MR(T)\geq MR(T^{\prime })$ for any tree *T* in $T_{\upsilon }^{\Delta }$. Let us denote the root vertex of $T^{\prime }$ by *ω* and the degree of *ω* in $T^{\prime }$ by Δ. According to Lemma 1, $T^{\prime }$ is a spider with its center located at *ω*. Assuming that *ω* is adjacent to *λ* leaves, the value of $MR(T^{\prime })$ can be computed as follows:

$$MR(T^{\prime })=\sum_{uv\in E^{T^{\prime }}}|\sqrt{d_{u}^{T^{\prime }}}-\sqrt{d_{v}^{T^{\prime }}}|=\lambda (\sqrt{\Delta }-1)+(\Delta -\lambda )(\sqrt{\Delta }-\sqrt{2})+(\Delta -\lambda )(\sqrt{2}-1)+(n-\Delta -2\lambda -1)(\sqrt{2}-\sqrt{2})=\Delta (\sqrt{\Delta }-1).$$

 □

Lemma 4 *If* b≥1 *, then function* $f(b)=b(\sqrt{b}-1)$ *is an increasing function.*

Proof If b≥1, then

$$f^{\prime }(b)=(\sqrt{b}-1)+\frac{b}{2\sqrt{b}}=\frac{3b-2\sqrt{b}}{2\sqrt{b}}>0.$$

 □

By Theorem 3 and Lemma 4, we obtain the next corollary.

Corollary 5 *If T is a tree of order υ, then*

$$MR(T)\geq 2(\sqrt{2}-1).$$

*Equality happens iff*$T=P_{\upsilon }$*.*

By Theorem 3, we can derive the following corollary:

Corollary 6 *Assume that T is a chemical tree in*$T_{\upsilon }^{\Delta }$*, where*3≤Δ≤4*. Then*$MR(T)\geq 3(\sqrt{3}-1)$*, when*Δ=3*, and*MR(T)≥4*, when*Δ=4*. In addition, the molecular tree T must have only one branching vertex to achieve these minimum values of the MR index.*

## 3 Lower bounds on unicyclic graphs with fixed order and maximum degree

**Figure 3:**
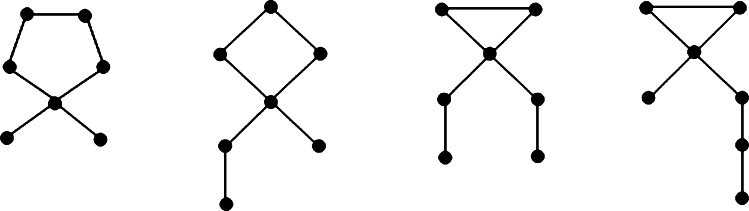
Cephalopod graphs with *υ* = 7 vertices and Δ = 4.

Throughout this section, let $U_{\upsilon }^{\Delta }$ represent the collection of all unicyclic graphs with *υ* vertices and a maximum degree of Δ. A cephalopod is defined as a unicyclic graph that has at most one vertex with a degree greater than 2 (see Fig. 3). In this context, a cycle with *υ* vertices is a specific type of cephalopod graph where all vertices have a degree of exactly two.

Lemma 7 *Suppose that*$U\in U_{\upsilon }^{\Delta }$*is a unicyclic graph in which no vertex of degree* Δ *lies on C, where C is the unique cycle of U. In addition, suppose that ω represents a vertex in U having degree* Δ *and ϑ denotes a vertex on C such that the distance* $d_{\left(\omega ,\vartheta \right)}^{U}$ *is minimized. If either* $d_{\vartheta }^{U}\geq 4$*, or there exists* $\eta \in V^{U}\setminus \{\omega ,\vartheta \}$ *for which* $d_{\eta }^{U}\geq 3$*, then the set* $U_{\upsilon }^{\Delta }$ *includes a graph* $U^{\prime }$ *such that* $MR(U)>MR(U^{\prime })$*.*

Proof Let *P* indicate the path in *U* between *ω* and *ϑ*. First assume the case where $\eta \in V^{U}\setminus \{\omega ,\vartheta \}$ is a vertex with $d_{\eta }^{U}\geq 3$. Let $T_{\eta }$ represent a rooted tree containing the greatest possible number of vertices joined to *η*.

If $\eta \notin V^{C}\cup V^{P}$, then according to Corollary 5, the tree $T_{\eta }$ can be transformed into a path $P_{\eta }$ that contains an equal number of vertices with the condition that $MR(T_{\eta })>MR(P_{\eta })$. Next, suppose that $U^{\prime }\in U_{\upsilon }^{\Delta }$ is made from the graph *U* by removing the sub tree $T_{\eta }$ and incorporating the path $P_{\eta }$. Thus, we have, $MR(U)>MR(U^{\prime })$.

Now consider the case where $\eta \in V^{C}$ (the case $\eta \in V^{P}$ can be proved in a similar manner) and suppose $\eta_{1},\eta_{2}$ be two vertices in the neighborhood $N_{\eta }^{U}\setminus V^{T_{\eta }}$, both of which lie on the cycle *C*. By applying Corollary 5, it is possible to transform the tree $T_{\eta }$ into a path $P_{\eta }$ that contains an equal number of vertices, satisfying the condition $MR(T_{\eta })\geq MR(P_{\eta })$. Assume that $U^{\prime }\in U_{\upsilon }^{\Delta }$ is made from the graph *U* by omitting $T_{\eta }$ and incorporating $P_{\eta }$. This modification leads to $MR(U)\geq MR(U^{\prime })$. Additionally, we have $d_{\eta }^{U^{\prime }}=3$. Now assume that $U^{\prime \prime }\in U_{\upsilon }^{\Delta }$ is made from $U^{\prime }$ by removing $P_{\eta }$ and incorporating the new path $\eta_{1}\;P_{\eta }\;\eta$. In this transformation, the degrees of vertices $\eta_{1}$ and $\eta_{2}$ remain unchanged, i.e., $d_{\eta_{1}}^{U^{\prime \prime }}=d_{\eta_{1}}^{U^{\prime }}$ and $d_{\eta_{2}}^{U^{\prime \prime }}=d_{\eta_{2}}^{U^{\prime }}$. Let's first assume the case where the length of $P_{\eta }$ is greater than 1. Then,

$$MR(U^{\prime })-MR(U^{\prime \prime })=\sum_{uv\in E^{U^{\prime }}}|\sqrt{d_{u}^{U^{\prime }}}-\sqrt{d_{v}^{U^{\prime }}}|-\sum_{uv\in E^{U^{\prime \prime }}}|\sqrt{d_{u}^{U^{\prime \prime }}}-\sqrt{d_{v}^{U^{\prime \prime }}}|=|\sqrt{3}-\sqrt{d_{\vartheta_{1}}^{U^{\prime }}}|+|\sqrt{3}-\sqrt{d_{\vartheta_{2}}^{U^{\prime }}}|+(\sqrt{3}-\sqrt{2})+(\sqrt{2}-1)-|\sqrt{2}-\sqrt{d_{\vartheta_{1}}^{U^{\prime \prime }}}|-|\sqrt{2}-\sqrt{d_{\vartheta_{2}}^{U^{\prime \prime }}}|=|\sqrt{3}-\sqrt{d_{\vartheta_{1}}^{U^{\prime }}}|+|\sqrt{3}-\sqrt{d_{\vartheta_{2}}^{U^{\prime }}}|+\sqrt{3}-1-\sqrt{d_{\vartheta_{1}}^{U^{\prime \prime }}}-\sqrt{d_{\vartheta_{2}}^{U^{\prime \prime }}}+2\sqrt{2}.$$

If $d_{\vartheta_{1}}^{U^{\prime }}\geq 3$ and $d_{\vartheta_{2}}^{U^{\prime }}\geq 3$, then

$$|\sqrt{3}-\sqrt{d_{\vartheta_{1}}^{U^{\prime }}}|+|\sqrt{3}-\sqrt{d_{\vartheta_{2}}^{U^{\prime }}}|=\sqrt{d_{\vartheta_{1}}^{U^{\prime \prime }}}+\sqrt{d_{\vartheta_{2}}^{U^{\prime \prime }}}-2\sqrt{3},$$

and $MR(U^{\prime })-MR(U^{\prime \prime })=2\sqrt{2}-\sqrt{3}-1≊0.09637>0$. If $d_{\vartheta_{1}}^{U^{\prime }}\geq 3$ and $d_{\vartheta_{2}}^{U^{\prime }}=2$ (the case $d_{\vartheta_{1}}^{U^{\prime }}=2$ and $d_{\vartheta_{2}}^{U^{\prime }}\geq 3$ is similar), then

$$|\sqrt{3}-\sqrt{d_{\vartheta_{1}}^{U^{\prime }}}|+|\sqrt{3}-\sqrt{d_{\vartheta_{2}}^{U^{\prime }}}|=\sqrt{d_{\vartheta_{1}}^{U^{\prime \prime }}}-\sqrt{3}+\sqrt{3}-\sqrt{2}=\sqrt{d_{\vartheta_{1}}^{U^{\prime \prime }}}-\sqrt{2},$$

and $MR(U^{\prime })-MR(U^{\prime \prime })=\sqrt{3}-1>0$. If $d_{\vartheta_{1}}^{U^{\prime }}=d_{\vartheta_{2}}^{U^{\prime }}=2$, then

$$|\sqrt{3}-\sqrt{d_{\vartheta_{1}}^{U^{\prime }}}|+|\sqrt{3}-\sqrt{d_{\vartheta_{2}}^{U^{\prime }}}|=2\sqrt{3}-2\sqrt{2},$$

and $MR(U^{\prime })-MR(U^{\prime \prime })=3\sqrt{3}-2\sqrt{2}-1>0$.

Now let's assume that the length of $P_{\eta }$ is one. In this case, we have

$$MR(U^{\prime })-MR(U^{\prime \prime })=\sum_{uv\in E^{U^{\prime }}}|\sqrt{d_{u}^{U^{\prime }}}-\sqrt{d_{v}^{U^{\prime }}}|-\sum_{uv\in E^{U^{\prime \prime }}}|\sqrt{d_{u}^{U^{\prime \prime }}}-\sqrt{d_{v}^{U^{\prime \prime }}}|=|\sqrt{3}-\sqrt{d_{\vartheta_{1}}^{U^{\prime }}}|+|\sqrt{3}-\sqrt{d_{\vartheta_{2}}^{U^{\prime }}}|+(\sqrt{3}-1)-|\sqrt{2}-\sqrt{d_{\vartheta_{1}}^{U^{\prime \prime }}}|-|\sqrt{2}-\sqrt{d_{\vartheta_{2}}^{U^{\prime \prime }}}|=|\sqrt{3}-\sqrt{d_{\vartheta_{1}}^{U^{\prime }}}|+|\sqrt{3}-\sqrt{d_{\vartheta_{2}}^{U^{\prime }}}|+\sqrt{3}-1-\sqrt{d_{\vartheta_{1}}^{U^{\prime \prime }}}-\sqrt{d_{\vartheta_{2}}^{U^{\prime \prime }}}+2\sqrt{2}.$$

Now in a similar manner, we have $MR(U^{\prime })-MR(U^{\prime \prime })>0$.

Next, assume that $d_{\vartheta }^{U}\geq 4$, with vertices $\vartheta_{1},\vartheta_{2},\vartheta_{3}\in N_{\vartheta }^{U}$, where $\vartheta_{1}$ and $\vartheta_{2}$ lie on the cycle *C* and $\vartheta_{3}$ lies on the path *P*. We can assign the degrees as $d_{\vartheta_{1}}^{U}=d_{\vartheta_{2}}^{U}=d_{\vartheta_{3}}^{U}=2$. Let $T_{\vartheta }$ represent a rooted tree having the greatest possible number of vertices joined to *ϑ*, ensuring that $\vartheta_{1},\vartheta_{2},\vartheta_{3}\notin V^{T_{\vartheta }}$. From Corollary 5, we can transform the tree $T_{\vartheta }$ into a path $P_{\vartheta }$ that contains the same number of vertices, for which $MR(T_{\vartheta })\geq MR(P_{\vartheta })$. Let $U^{\prime }$ be a graph in $U_{\upsilon }^{\Delta }$ made from *U* by removing the path $T_{\vartheta }$ and incorporating the path $P_{\vartheta }$. Thus, we have $MR(U)\geq MR(U^{\prime })$ and $d_{\vartheta }^{U^{\prime }}=4$. Now, assume that $U^{\prime \prime }$ is a graph in $U_{\upsilon }^{\Delta }$ constructed from $U^{\prime }$ by removing $P_{\vartheta }$ and incorporating the new path $\vartheta_{1}\;P_{\vartheta }\;\vartheta$. By this transformation the degrees of the vertices $\vartheta_{1}$, $\vartheta_{2}$, and $\vartheta_{3}$ remain unchanged, i.e., $d_{\vartheta_{1}}^{U^{\prime \prime }}=d_{\vartheta_{1}}^{U^{\prime }}=d_{\vartheta_{2}}^{U^{\prime \prime }}=d_{\vartheta_{2}}^{U^{\prime }}=d_{\vartheta_{3}}^{U^{\prime \prime }}=d_{\vartheta_{3}}^{U^{\prime }}=2$. If $P_{\vartheta }$ has a length more than 1, then

$$MR(U^{\prime })-MR(U^{\prime \prime })=\sum_{uv\in E^{U^{\prime }}}|\sqrt{d_{u}^{U^{\prime }}}-\sqrt{d_{v}^{U^{\prime }}}|-\sum_{uv\in E^{U^{\prime \prime }}}|\sqrt{d_{u}^{U^{\prime \prime }}}-\sqrt{d_{v}^{U^{\prime \prime }}}|=3(\sqrt{4}-\sqrt{2})+(\sqrt{4}-\sqrt{2})+(\sqrt{2}-1)-3(\sqrt{3}-\sqrt{2})=7-3\sqrt{3}>0,$$

whereas otherwise,

$$MR(U^{\prime })-MR(U^{\prime \prime })=\sum_{uv\in E^{U^{\prime }}}|\sqrt{d_{u}^{U^{\prime }}}-\sqrt{d_{v}^{U^{\prime }}}|-\sum_{uv\in E^{U^{\prime \prime }}}|\sqrt{d_{u}^{U^{\prime \prime }}}-\sqrt{d_{v}^{U^{\prime \prime }}}|=3(\sqrt{4}-\sqrt{2})+(\sqrt{4}-1)-3(\sqrt{3}-\sqrt{2})=7-3\sqrt{3}>0.$$

 □

The argument for the next lemma follows an analogous approach to that outlined in Lemma 7 and is therefore omitted for brevity.

Lemma 8 *Assume that U is a graph in*$U_{\upsilon }^{\Delta }$*that contains a vertex ω having a degree of* Δ*, and that ω is situated on the cycle of U. If a vertex* $\eta \in V^{U}\setminus \{\omega \}$ *exists such that* $d_{\eta }^{U}\geq 3$*, then it follows that the set* $U_{\upsilon }^{\Delta }$ *includes a graph* $U^{\prime }$ *for which* $MR(U)>MR(U^{\prime })$*.*

Lemma 9 *Let U be a graph in*$U_{\upsilon }^{\Delta }$*that meets the condition that none of its vertices with degree* Δ *are on the cycle of U. Then, the set* $U_{\upsilon }^{\Delta }$ *contains a graph* $U^{\prime }$ *in which the vertex with the degree* Δ *is located at the cycle of* $U^{\prime }$*, and it holds that* $MR(U^{\prime })<MR(U)$*.*

Proof Assume that *ω* represents a vertex of the graph *U* with $d_{\omega }^{U}=\Delta$, and *C* denotes the unique cycle of this graph. Suppose *ϑ* is a vertex on *C* that is closest to *ω* among all other vertices on the cycle *C* with a degree greater than 2. Define the path from *ω* to *ϑ* as *P*. From Lemma 7, we have $d_{\vartheta }^{U}=3$ and for each vertex $\eta \in V^{U}\setminus \{\omega ,\vartheta \}$, it follows that $d_{\eta }^{U}\leq 2$. Define the neighborhoods of *ω* and *ϑ* in *U* to be $N_{\omega }^{U}=\{\omega_{1},\omega_{2},...,\omega_{\Delta }\}$ and $N_{\vartheta }^{U}=\{\vartheta_{1},\vartheta_{2},\vartheta_{3}\}$, where both vertices $\omega_{\Delta }$ and $\vartheta_{1}$ lie on the path *P*, while the vertices $\vartheta_{2}$ and $\vartheta_{3}$ lie on the cycle *C*.

Let $T_{\omega }$ be the rooted tree that maximizes the number of vertices attached to *ω*, ensuring that $V^{T_{\omega }}\cap P=\{\omega \}$. Using Lemma 1, this tree can be transformed into a spider graph, which we denote as $T_{\omega }^{\prime }$ in such a way that $T_{\omega }^{\prime }$ retains the same number of vertices, has its center at vertex *ω*, and satisfies the inequality $MR(T_{\omega }^{\prime })\leq MR(T_{\omega })$. Let $U^{\prime }\in U_{\upsilon }^{\Delta }$ be obtained from *U* by removing $T_{\omega }$ and incorporating $T_{\omega }^{\prime }$. Applying Lemma 1, we conclude that $MR(U^{\prime })\leq MR(U)$.

Denote by $u_{1},\ldots ,u_{\Delta -1}$ the pendant vertices of $T_{\omega }^{\prime }$ and by $z_{1},\ldots ,z_{t}$ the vertices of $T_{\omega }^{\prime }$ that have degree two. Let $U^{\prime \prime }\in U_{\upsilon }^{\Delta }$ be constructed from $U^{\prime }$ by removing the vertices in $V^{T_{\omega }^{\prime }}\setminus \{\omega_{\Delta }\}$ and incorporating the edges in $\left\{\vartheta u_{1},\ldots ,\vartheta u_{\Delta -3}\right\}$ along with the path $\omega_{\Delta }\omega z_{1}\ldots z_{t}u_{\Delta -2}u_{\Delta -1}$. In the case that $\vartheta \omega \in E^{U}$, we have

$$MR(U^{\prime })-MR(U^{\prime \prime })=\sum_{uv\in E^{U^{\prime }}}|\sqrt{d_{u}^{U^{\prime }}}-\sqrt{d_{v}^{U^{\prime }}}|-\sum_{uv\in E^{U^{\prime \prime }}}|\sqrt{d_{u}^{U^{\prime \prime }}}-\sqrt{d_{v}^{U^{\prime \prime }}}|=(\sqrt{\Delta }-\sqrt{3})+(\Delta -1)(\sqrt{\Delta }-1)+2(\sqrt{3}-\sqrt{2})-(\Delta -3)(\sqrt{\Delta }-1)-3(\sqrt{\Delta }-\sqrt{2})-(\sqrt{2}-1)=\sqrt{3}-1>0,$$

whereas otherwise,

$$MR(U^{\prime })-MR(U^{\prime \prime })=\sum_{uv\in E^{U^{\prime }}}|\sqrt{d_{u}^{U^{\prime }}}-\sqrt{d_{v}^{U^{\prime }}}|-\sum_{uv\in E^{U^{\prime \prime }}}|\sqrt{d_{u}^{U^{\prime \prime }}}-\sqrt{d_{v}^{U^{\prime \prime }}}|=(\sqrt{\Delta }-\sqrt{2})+(\Delta -1)(\sqrt{\Delta }-1)+3(\sqrt{3}-\sqrt{2})-(\Delta -3)(\sqrt{\Delta }-1)-3(\sqrt{\Delta }-\sqrt{2})-(\sqrt{2}-1)=3\sqrt{3}-2\sqrt{2}-1>0.$$

Then the proof is complete. □

The following outcome represents our second main theorem.

Theorem 10 *Let*$U\in U_{\upsilon }^{\Delta }$*. Then,*

$$MR(U)\geq \Delta (\sqrt{\Delta }-1)-2(\sqrt{2}-1),$$

*with equality iff U is a cephalopod.*

Proof Given that $U^{\prime }$ is a graph in $U_{\upsilon }^{\Delta }$ with $MR(U)\geq MR(U^{\prime })$ for any $U\in U_{\upsilon }^{\Delta }$, and assuming that *ω* is a vertex with $d_{\omega }^{U^{\prime }}=\Delta$, we can conclude by Lemma 7, Lemma 8, and Lemma 9 that $U^{\prime }$ is a cephalopod graph whose center is at *ω*. Furthermore, suppose that among the neighbors of *ω*, there exist *λ* leaves. Hence,

$$MR(U^{\prime })=\sum_{uv\in E^{U^{\prime }}}|\sqrt{d_{u}^{U^{\prime }}}-\sqrt{d_{v}^{U^{\prime }}}|=\lambda (\sqrt{\Delta }-1)+(\Delta -\lambda )(\sqrt{\Delta }-\sqrt{2})+(\Delta -\lambda -2)(\sqrt{2}-1)+(n-\Delta -2\lambda +1)(\sqrt{2}-\sqrt{2})=\Delta (\sqrt{\Delta }-1)-2(\sqrt{2}-1).$$

 □

In a manner similar to the Lemma 4, we arrive at the result below.

Lemma 11 *If*b≥1*, then the function*$f(b)=b(\sqrt{b}-1)-2(\sqrt{2}-1)$*is an increasing function.*

The following corollary is directly derived from Theorem 10 and Lemma 11.

Corollary 12 *For each unicyclic graph U of order υ, we have*MR(U)≥0*. In addition,*MR(U)=0*iff*$U=C_{\upsilon }$*.*

## 4 Discussion and conclusion

The focus of this paper is on the *MR* index, which is a relatively under-studied discrete Adriatic index. The *MR* index has not only demonstrated significant predictive capabilities in evaluations performed by the IAMC, but it also serves as a useful means for quantifying the irregularity extent in graphs and/or molecular graphs. To encourage researchers to further investigate the *MR* index, we have considered here some of its extremal properties for trees and unicyclic graphs with a specified number of vertices and maximum vertex degree. In particular, we have proven that within the specified family of trees, the minimum value of the *MR* index is $\Delta (\sqrt{\Delta }-1)$, achieved by spider graphs. Meanwhile, within the specified family of unicyclic graphs, the minimum value of the *MR* index is $\Delta (\sqrt{\Delta }-1)-2(\sqrt{2}-1)$, which occurs in cephalopod graphs. Since the *MR* index is regarded as an effective tool for forecasting the enthalpy of vaporization, along with the standard enthalpy of vaporization for isomers of octane, our findings could contribute to advancements in QSAR and QSPR studies related to these structures. Furthermore, as numerous classes of chemical graphs associated with molecular compounds can be modeled as trees or unicyclic graphs, our findings may facilitate a more comprehensive examination of the physicochemical properties of these compounds. The minimal bicyclic, tricyclic, and multi-cyclic graphs with a specified order and fixed maximum degree concerning the *MR* index can be effectively analyzed using the methods outlined in this paper.

## Funding

This research did not receive any external financial or non-financial assistance.

## CRediT authorship contribution statement

**Nasrin Dehgardi:** Writing – original draft, Visualization, Validation, Methodology, Investigation. **Mahdieh Azari:** Writing – review & editing, Validation, Methodology, Investigation, Conceptualization. **Yilun Shang:** Writing – review & editing, Validation, Resources, Investigation.

## Declaration of Competing Interest

The authors declare the following financial interests/personal relationships which may be considered as potential competing interests: Yilun Shang, one of the coauthors of the present paper, is a section editor of the journal Heliyon where the present paper is being submitted. Other authors declare that they have no known competing financial interests or personal relationships that could have appeared to influence the work reported in this paper.

## Data Availability

Data sharing not applicable to this article as no datasets were generated or analyzed during the current study.
